# Flow Cytometry Analysis in Breast Implant-Associated Anaplastic Large Cell Lymphoma: Three Case Reports

**DOI:** 10.3390/ijms25063518

**Published:** 2024-03-20

**Authors:** Veronica Davanzo, Alessandra Falda, Paola Fogar, Kathrin Ludwig, Jenny Zuin, Maria Cristina Toffanin, Marco Pizzi, Angelo Paolo Dei Tos, Daniela Basso

**Affiliations:** 1 Laboratory Medicine Unit, Biomedical Sciences Department—DSB, University of Padova, 35128 Padova, Italy; 2 Laboratory Medicine Unit, Integrated Diagnostic Services—DIDAS, Padova University Hospital, 35128 Padova, Italyjenny.zuin@aopd.veneto.it (J.Z.); daniela.basso@unipd.it (D.B.); 3 Surgical Pathology and Cytopathology Unit, Department of Medicine—DIMED, Padova University Hospital, 35128 Padova, Italyangelo.deitos@unipd.it (A.P.D.T.); 4 Department of Breast Surgery, Veneto Institute of Oncology IOV IRCCS, 35128 Padova, Italy; 5 Surgical Pathology and Cytopathology Unit, Department of Medicine—DIMED, University of Padova, 35128 Padova, Italy; 6 Laboratory Medicine Unit, Department of Medicine—DIMED, University of Padova, 35128 Padova, Italy

**Keywords:** BIA-ALCL, CD30, flow cytometry, stability, morphology, immunohistochemistry

## Abstract

Breast Implant-Associated-Anaplastic Large Cell Lymphoma (BIA-ALCL) is a rare T-cell non-Hodgkin lymphoma associated with breast prosthetic implants and represents a diagnostic challenge. The National Comprehensive Cancer Network (NCCN) guidelines, updated in 2024, recommend for diagnosis an integrated work-up that should include cell morphology, CD30 immunohistochemistry (IHC), and flow cytometry (FCM). CD30 IHC, although the test of choice for BIA-ALCL diagnosis, is not pathognomonic, and this supports the recommendation to apply a multidisciplinary approach. A close collaboration between pathologists and laboratory professionals allowed the diagnosis of three BIA-ALCLs, presented as case reports, within a series of 35 patients subjected to periprosthetic effusions aspiration from 2018 to 2023. In one case, rare neoplastic cells were identified by FCM, and this result was essential in leading the anatomopathological picture as indicative of this neoplasm. In fact, the distinction between a lymphomatous infiltrate from reactive cells may be very complex in the cytopathology and IHC setting when neoplastic cells are rare. On the other hand, one limitation of FCM analysis is the need for fresh samples. In this study, we provide evidence that a dedicated fixative allows the maintenance of an unaltered CD30 expression on the cell surface for up to 72 h.

## 1. Introduction

BIA-ALCL is a T-cell non-Hodgkin lymphoma (NHL) that develops around breast implants, now emerging also in other implant types [1]. NHLs in the breast represent <1% of breast neoplasms, and 10% of these are T-cell lymphomas. BIA-ALCL was identified in 1997, but it was only after a communication from the Food and Drug Administration (FDA) in 2011 that interest in this pathology increased [2].

In 2016, the 4th edition of the World Health Organization (WHO) identified this lymphoma as a provisional entity, and in the following year the NCCN drafted the first guidelines for diagnostic and therapeutic workflow [3,4]. In 2022, the WHO updated this condition to a definitive entity, and the International Consensus Classification (ICC) also recognized BIA-ALCL as a fully defined disease [5,6]. Between 5 and 10 million women worldwide have breast implants, with over 1.5 million women receiving them annually [7]. The incidence of BIA-ALCL varies among study cohorts, with estimates ranging from 1:354 to 1:37,000. This most likely depends on different management and adherence to national and/or international registries on BIA-ALCL by different countries, as well as socio-economic and cultural reasons that involve different uses of prosthetic implants across the world [2,8].

The distribution of the disease is equal among cosmetic and reconstructive mastectomy patients.

BIA-ALCL is typically a slow-growing, localized disease with a favorable prognosis when patients undergo surgical removal. The overall survival rate is estimated at 94% and 91% at 3 and 5 years, respectively [9]. As of June 2023, there were 1264 global cases and 63 deaths. A majority of the cases involved exposure to textured surface implants [7,10,11,12,13]. NCCN data suggest that bilateral cases could account for 2–4% of all BIA-ALCL diagnoses [9].

Consensus implicates that the induction of a chronic inflammatory state, especially in a genetic susceptibility background (i.e., genetic variants of *DNMT3A*, *JAK-STAT3* pathway, and *TP53*), underlies malignant transformation [11,14,15,16,17].

The most shared theory considers the implanted prosthesis per se and superimposed infections as chronic antigenic stimuli evoking an excessive inflammatory response [18]. Culture studies reveal that *Staphylococcus aureus* and *Enterobacter* spp. are the microorganisms most frequently involved in implant effusions. In rare cases, colonization of *Candida albicans* and *Aspergillus niger* or viral infections, have been observed [11,19]. However, BIA-ALCL cells present a stronger response to lipopolysaccharide (LPS) stimulation than other bacterial agents [11,16]. Unlike smooth surface implants, textured implants have a larger surface area and consequently might favor the presence of a greater number of bacteria that can form a biofilm on the prosthesis [16,20]. This aspect could contribute to the increased incidence of BIA-ALCL in patients with textured prosthesis.

**Figure 1:**
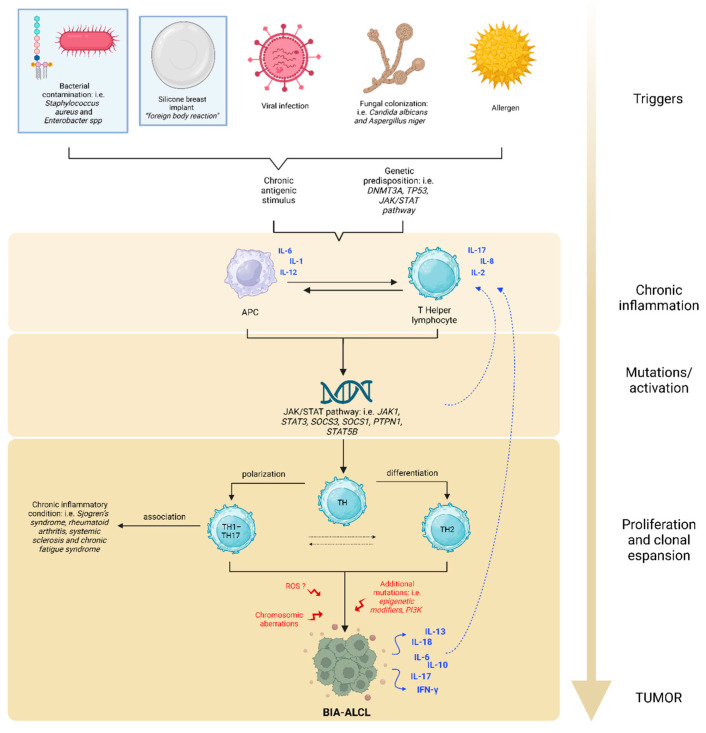
Possible etiopathogenesis of BIA-ALCL. The oncogenesis of BIA-ALCL is a multistep process, similar to that of solid tumors, but is still not completely understood. The main triggers may be LPS, prosthetic material, oncogenic viruses, fungal colonization, or allergens. These inputs result in the activation of macrophages as antigen presenting cells (APCs). The consequent activation of naïve T cells sustains chronic inflammation, resulting in aberrant STAT3 signaling, which feeds the pro-carcinogenic inflammatory microenvironment. As long as the immune cascade remains activated, the risk of DNA alteration in overstimulated cells increases, inducing the onset of *JAK/STAT3* activating mutations. Excess STAT3 leads to differentiation of Th1/Th17 and Th2 lymphocytes and, along with other additional alterations, causes clonal expansion of T cells. Other chromosomic aberrations and ROS damages may contribute to the neoplastic process. BIA-ALCL cells secrete many cytokines that self-enhance the inflammatory state. Among all, IL-13, a Th2 interleukin characteristic of the allergic phenomenon, is also produced.

As illustrated in Figure 1, the processes suggested for the development of BIA-ALCL involve the presence of an antigenic trigger (LPS or other bacterial antigens, prosthetic material as a foreign body, potentially oncogenic viruses or allergens) which stimulates macrophages in their antigen presenting function. Subsequent interaction with naïve helper T cells sustains a state of chronic inflammation.

Consequentially, abnormal STAT3 stimulation persists and it might be associated with *STAT3* mutations in a self-perpetuating cycle. In agreement with this concept, Quesada and Di Napoli observed several genetic variants of the *JAK-STAT3* pathway by targeted next-generation sequencing (NGS) of BIA-ALCL [14,21]. Excess STAT3 promotes the differentiation of Th1/Th17 and Th2 lymphocyte phenotypes, leading to uncontrolled clonal expansion of T cells and the formation of BIA-ALCL. Genetic variants in the oncosuppressor genes *TP53* and *DNMT3A* have also been documented [11,14,15,16,17,22].

Some authors hypothesize that a release of chemically active compounds from the prosthesis can induce the production of reactive oxygen species (ROS), which in turn contribute to the onset of chromosomal aberrations favoring the development of the neoplasm [18,23].

In fact, the loss of tumor suppressor genes has also been reported as a consequence of chromosomal alterations, deletions being the most frequently found [18].

This multistep pathogenesis may explain the long latency time (average of 10 years) between prosthesis implantation and the development of lymphoma [9,20].

Moreover, Kadin [24] demonstrated eosinophil infiltration in lymphoma tissues of BIA-ALCL and positive staining for IL-13 in anaplastic lymphoma cells. The presence of eosinophils and mast cells in tumor tissue is indicative of allergic inflammation associated with IL-13. The allergic nature of the antigenic stimulus was reported again by Kadin [24], who observed the production of the Th2 cytokine IL-13 and a variably expressed Th2 transcription factor GATA3.

Interestingly, constitutively active STAT3 induces a Th17 phenotype, the expression of Th2 associated cytokines, and transcription factor, further comports the role of allergic inflammation.

In cell lines of BIA-ALCL, the activation of the *JAK/STAT3* signaling pathway and expression of cytotoxic molecules were observed, comparable to the findings in systemic ALK+ ALCL, particularly the Th17 related upregulation genes [10,25].

Studies of Kadin and Di Napoli [14,24] suggest that BIA-ALCL tumors likely derive from CD4+ memory-activated T cells with features of Th1/Th17.

Interestingly, Th1/Th17 cells are antigen-driven memory T cells implicated in different chronic inflammatory conditions, such as rheumatoid arthritis, psoriasis, and cancer. This phenomenon could support the increased risk of developing autoimmune and rheumatic diseases (i.e., Sjogren’s syndrome, rheumatoid arthritis, systemic sclerosis, and chronic fatigue syndrome) in patients with breast implants [26]. Some studies show an increased risk of positive immunological markers in women with symptoms of breast implant illness (for example antinuclear antibodies, anticardiolipin, anti-rheumatoid factor antibodies and anti-dsDNA). This data also suggests that breast implants may trigger an immune response [21].

The most frequent symptom of BIA-ALCL (in 60–90% of cases) is a large spontaneous periprosthetic fluid collection that appears at least one year after implantation. Periprosthetic effusion occurs in about 0.1% of all breast implant procedures. It can be caused by implant rupture, hematoma, silicone leakage, trauma, infection, or other, with BIA-ALCL being the cause in about 10% of cases. Around 30% of patients show up with a tumor mass, with or without effusion. Rarely, patients exhibit regional lymphadenopathy, usually in the axillary region. Finally, there are a few instances where BIA-ALCL is found unexpectedly, during surgery for unrelated reasons [7,9,11,27,28]. Fine needle aspiration (FNA) or mass biopsy is indicated in case of suspicious effusion or mass, once confirmed by ultrasonography or magnetic resonance imaging [29].

The cytopathological analysis is fundamental in the differential diagnosis between reactive and neoplastic conditions, including BIA-ALCL [7,29].

Moreover, according to the NCCN [9], FCM is now considered useful in the diagnostic work-up, and this is particularly true in a morphological suspicion for lymphoma.

Prosthesis removal and total capsulectomy is an effective treatment for confirmed BIA-ALCL. Computed tomography or magnetic resonance imaging scans are useful for studying soft tissue when ultrasound is inconclusive, and may be helpful in planning surgery if a mass is present.

For advanced stages (II, III, or IV), the first-line treatment should focus on chemotherapy and radiotherapy, based on the guidelines for systemic ALCL. Immunotherapy (brentuximab vedotin) should be considered and is administered either as monotherapy or in combination as first-line or second-line treatment [9,29].

## 2. Detailed Case Description

From 2018 to 2023, 37 breast peri-implant effusion samples from 35 patients were evaluated by the Laboratory Medicine Unit of the University-Hospital of Padova for suspicion of BIA-ALCL. Samples were collected at the Veneto Oncology Institute (IOV), which is recognized by the Italian Ministry of Health as one of the reference centers for the clinical management of patients diagnosed with BIA-ALCL [30]. In three of these patients, the diagnosis of BIA-ALCL was established as described below.

### 2.1. Case #1

The first case was partially presented by the authors in a national journal of the laboratory medicine as a single case report [31].

**Figure 2:**
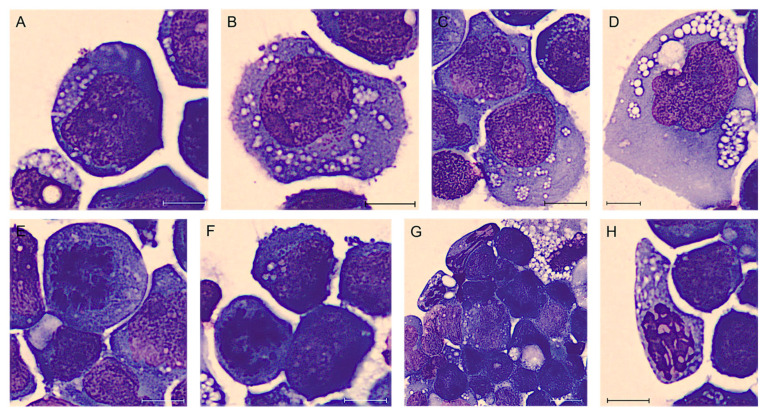
BIA-ALCL of Case #1 periprosthetic effusion after May–Grünwald Giemsa staining (100× magnification). Atypical lymphoid cells of different sizes, often large, with cytoplasm variably basophilic frequently containing many small vacuoles also in aggregate and noticeable nucleoli (**A**–**D**). Sometimes they are clearly anaplastic with multinucleated nuclei resembling Reed–Sternberg cells (**D**). It is also common to observe unusual mitoses (**E**,**F**) and apoptotic cells (**G**,**H**). Scale bar: 10 µm.

In December 2021, a 51-year-old woman was admitted for suspected left periprosthetic hematoma. The patient’s clinical history recorded a previous infiltrating lobular carcinoma of the left breast staged G2 (diagnosed in January 2010) treated with radical mastectomy and unilateral left prosthesis implantation (May 2011). From August 2021, the woman complained about the presence of a periprosthetic flap, not associated with fever or other signs of inflammation, and therefore she underwent a revision of the pocket in December 2021. During this procedure, a cold periprosthetic effusion was aspirated, which was sent for FCM and immunohistochemical analysis. The morphological evaluation of the yellow–amber periprosthetic fluid was performed by human digital cell morphology (Cella Vision DM96, Sysmex, Kobe, Japan) and showed the presence of large anaplastic cells. Those cells presented a high nucleus/cytoplasm ratio with an abundant basophilic and granular cytoplasm, pleomorphic nuclei with vesicular or dense chromatin, and prominent nucleoli (Figure 2) [31].

**Figure 3:**
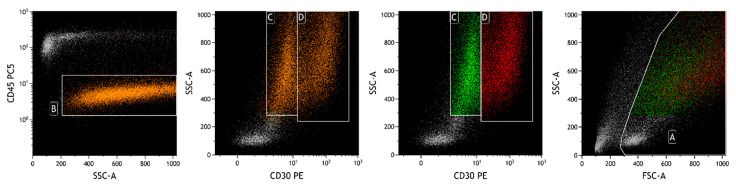
FCM plots of Case #1 of BIA-ALCL. A: live cells; B: neoplastic cells; C and D: neoplastic cells divided according to the intensity of CD30 expression. A clear heterogeneity in the intensity of CD30 (in orange) is observed on CD30-PE/SSC plot, detecting two apparent subpopulations of tumor cells (green and red cells) with different distribution on Forward Scatter (FSC) and Side Scatter (SSC).

Flow cytometric analysis was performed with a Navios Ex flow cytometer and data were analyzed using Kaluza analysis software v. 2.1 (Beckman Coulter, Brea, CA, USA). A lymphoid population with an abnormal phenotype was observed, raising the suspicion of BIA-ALCL. These neoplastic cells represented 74% of total cellularity, had increased physical parameters, an aberrant expression of CD30, a strong positivity for CD2 and a partial one for CD4, and loss of expression of the leukocyte common antigen CD45 and of CD3, CD5, CD7, T-associated antigens [31]. In agreement with previous findings [32,33,34,35,36], we observed a bimodal CD30 intensity (Figure 3), but no variations in the expression of the other studied markers.

**Figure 4:**
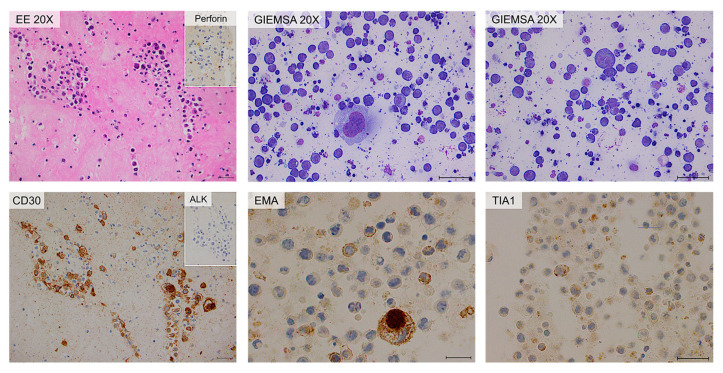
Cytology and immunohistochemistry of Case #1. The periprosthetic capsule (left panels) and cytoinclusions are shown. Periprosthetic capsule: hematic and fibrinous material containing large atypical neoplastic cells. Immunohistochemically, the neoplastic cells are diffusely positive for CD30 and TIA-1 stains. Cytoinclusions: evident presence of numerous atypical lymphocytes of heterogeneous dimensions, even large, positive for EMA (partial) and TIA-1 (focal) stains. Scale bar: 20 µm.

IHC of cytoinclusion also highlighted an abnormal T phenotype of tumor cells: they were positive for CD30, CD4, CD2, CD3, CD15, EMA, and TIA-1 stains and negative for ALK-1, CD5, CD7, CD20, EBER and PAX5 stains. Lastly, immunohistochemistry of the periprosthetic capsule, obtained during the revision of the pocket, demonstrated that all the neoplastic cells were CD30 and Perforin positive, and they expressed an incomplete T-cell phenotype (Figure 4).

The patient underwent surgical excision of the prosthesis with capsulectomy. No relapses were recorded in the follow-up.

### 2.2. Case #2

**Figure 5:**
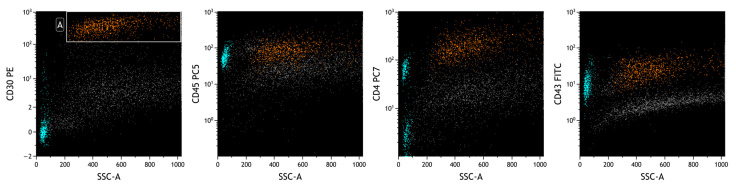
FCM plots of Case #2 of BIA-ALCL. The lymphoid neoplastic population (A, in orange) presents a different phenotype from the first patient’s. Lymphocytes can also be seen (in heavenly), mainly represented by positive CD4 T lymphocytes. A different intensity of CD30 expression between reactive cells (lymphocytes) and neoplastic cells is observed on the CD30-PE/SSC plot.

**Figure 6:**
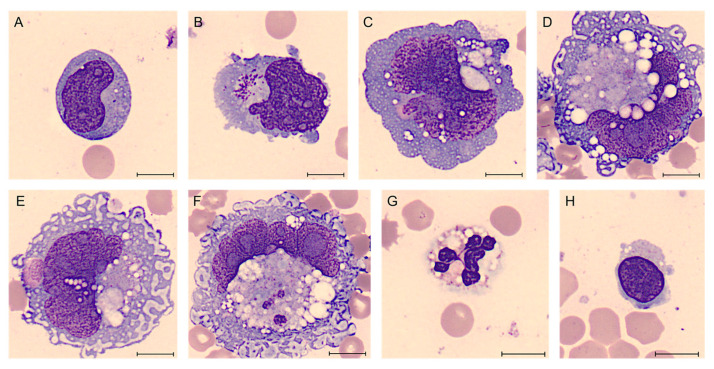
BIA-ALCL of Case #2 periprosthetic effusion after May-Grünwald Giemsa staining (100× magnification). Atypical lymphoid cells of different sizes, with pale cytoplasm, frequently containing many small vacuoles (**A**–**D**). Cells are clearly anaplastic with multinucleated nuclei (**A**–**C**) and sometimes they have the appearance of “signet ring cells” (**D**–**F**). The reactive cells are represented by neutrophils and predominantly lymphocytes, in a frankly bloody material (**G**,**H**). Scale bar: 10 µm.

**Figure 7:**
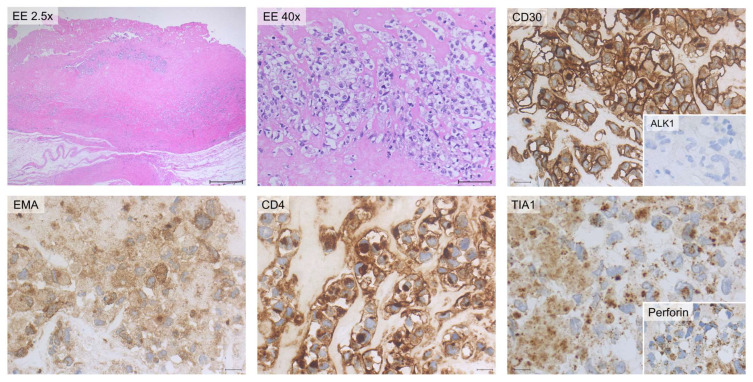
Histology and immunohistochemistry of Case #2. Breast parenchyma and periprosthetic capsule are shown. The presence of sheets of large atypical blasts with irregular nuclear contours, coarse chromatin and evident nucleoli can be observed. The lymphoid cells are surrounded by fi-brinous material with only scattered atypical large neoplastic cells. Immuno-histochemically, the neoplastic cells are diffusely positive for CD30, CD4, EMA, TIA-1, and Perforin stains while they are consistently negative for ALK-1 stain. Scale bar of the top left image: 0.5 cm; other scale bars: 20 µm.

A 34-year-old woman underwent additive mastoplasty for aesthetic purposes in 2011. After ten years, both prostheses were removed due to periprosthetic effusion and rupture of the left implant. Three months after surgery (February 2022), she was admitted to the IOV for recurrence of effusion, confirmed with a breast palpatory examination and echography. Her left breast was deformed by a not very compressible collection, without signs of inflammation, mass, lymphadenopathy, or any other lesions. An FNA was performed and the yellow–orange fluid was analyzed as previously described. Flow cytometric analysis showed the presence of an atypical lymphoid population, accounting for 50% of total cellularity, with increased physical parameters, even higher than those of the patient described above, an aberrant expression of CD30, a strong positivity for CD45, CD4, CD43, and loss of expression of the other T-associated antigens (CD3, CD2, CD5, CD7, and CD8) (Figure 5). At morphology, the cells were four to five times larger than a mature lymphocyte, with a much more eosinophilic and granular cytoplasm. In this case, the cytoplasmic vacuoles were so abundant that, combining together, gave the neoplastic cells a signet ring appearance (Figure 6) [10]. Moreover, cellular outlines demonstrate cytoplasmic fragmentation. This morphology has also been described by Hu et al. [36]. Immunohistochemically, the neoplastic cells were diffusely positive for CD30, CD4, CD15, EMA, TIA-1, and Perforin. ALK-1, CD3, CD20, and PAX5 stains were consistently negative (Figure 7).

Like the first woman, this patient had no relapses.

### 2.3. Case #3

In September 2022 a 61-year-old woman was admitted for suspected right periprosthetic effusion, developed about six years after additive mastoplasty due to carcinoma of the right breast (diagnosed in 2012, prosthesis implantation in January 2016).

The yellow–orange periprosthetic effusion aspirated during the revision of the pocket was analyzed as previously described.

**Figure 8:**
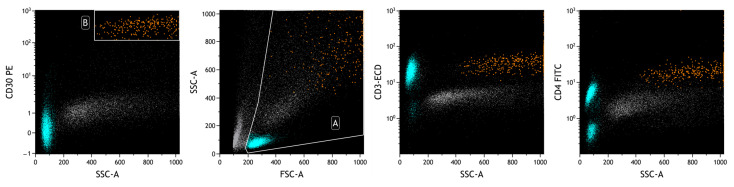
FCM plots of Case #3 of BIA-ALCL. A: live cells; B: neoplastic cells. The prevalence of T lymphocytes in the analyzed effusion is evident (in heavenly). The physical parameters do not allow the delimitation of the neoplastic population (in orange), which is identified with surface markers, including those highlighted in the plots. A different intensity of CD30 expression is observed between reactive cells (lymphocytes) and neoplastic cells.

Flow cytometric analysis revealed the presence of a small lymphoid population, accounting for 4% of total cellularity, with increased physical parameters, a strong positivity for CD30 and CD4, CD45 moderate, and loss of expression of T-associated antigens (CD2, CD3, CD5, and CD7). This abnormal phenotype raised the suspicion of BIA-ALCL (Figure 8).

Regarding morphology, the presence of rare elements of likely large lymphoid nature with mono/multilobed nucleus, also Sternbergoid and anaplastic, in a hypocellular and amorphous context could be observed.

**Figure 9:**
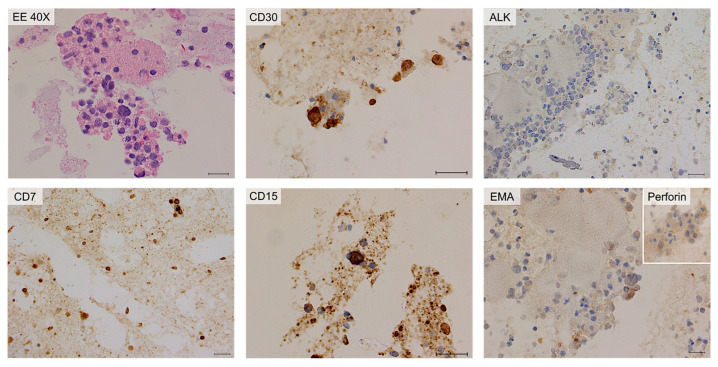
Cytology and immunohistochemistry of Case #3 cytoinclusions. Predominantly amorphous, sparsely cellular material including rare neutrophilic granulocytes, macrophages, small lymphocytes, and isolated atypical large elements. Immunohistochemically, the neoplastic cells are positive for CD30 and CD15 stains and negative for ALK-1 and EMA stains. CD7 stain is weakly/partially positive; Perforin is partially positive. Scale bar: 40 µm.

The cytological examination revealed the presence of rare cells, not suggesting per se the presence of lymphoproliferative disorders. Only after knowledge of the FCM results, targeted immunohistochemical analysis was performed, which confirmed the diagnosis of BIA-ALCL. Atypical cells were positive for CD30, CD4, CD15, and Perforin and negative for ALK-1, CD3, CD8, CD2, EMA, CD20, and PAX5. CD7 was weakly/partially positive (doubtful positivity) (Figure 9).

The combination of these techniques was instrumental to avoid additional invasive test in to the patient. No relapse has occurred at the time of the manuscript submission.

In all three cases, the reactive, non-neoplastic cellularity of the effusions was mainly represented by T lymphocytes (with a reduced CD4/CD8 ratio) and partly by monocytes-macrophages, with the percentage of neutrophilic granulocytes less than 5%. These findings are consistent with the concept that this pathology is one of typically chronic effusions [19,20,35].

Table 1 reports the flow cytometric and immunohistochemical characteristics of the three BIA-ALCL cases.

## 3. Materials and Methods

BIA-ALCL is a disease with excellent prognosis when patients undergo surgical excision. For this reason, its recognition is crucial. This will be even more so in the coming years, due to the increasing number of breast implants used worldwide [7].

Ideally, to avoid cellular degradation, the effusion should be processed immediately. Since cell damage depends on several variables, such as enzymatic activity and the presence of bacteria, it is generally agreed among laboratories that the sample should be immediately analyzed, or refrigerated for a maximum of 24 h before processing [19].

A limit of FCM is the analysis of preferential fresh material to avoid analytical artifacts (i.e., not specificity of fluorescence signal due to excessive debris). On the other hand, FCM has a high capacity to identify rare neoplastic cells, like CD30-positive ones. This characteristic is useful for an integrated report with cytology and IHC. For this reason, we studied the positivity of CD30 over time.

To our knowledge, there have been no published reports on the stability of CD30 by FCM in BIA-ALCL.

Not having material from BIA-ALCL, we simulated the matrix using fixed lysed peripheral blood and peripheral blood mononuclear cells (PBMCs) combined with ascitic fluids. We also studied the correlation between the expression of CD30 and 7-aminoactinomycin D (7-AAD) in unfixed PBMCs over time. The markers and their specifications used in FCM and IHC analysis are described in the respective Supplementary Tables (Tables S1 and S2).

## 4. Results

A whole blood sample (tube anticoagulated with K3-EDTA) from a patient with infectious mononucleosis was analyzed (total leukocytes: 15.20 × 10^9^/L; lymphocytes: 8.01 × 10^9^/L; T lymphocytes: 96% of total lymphocytes; T cytotoxic lymphocytes: 55%). We chose this type of sample because this viral infection typically involves strong lymphocyte activation, with consequent expression of the CD30 antigen on T lymphocytes.

A second type of sample was represented by lymphocytes stimulated with phytohemagglutinin (PHA). It is a protein that stimulates T-cell activation and proliferation and induces CD30 expression on T cells. In our experiments, PBMCs were first isolated from a healthy patient and then exposed to PHA [4 μg/mL] (PHA-L, ROCHE, Basel, Switzerland) for 48 h at 37 °C in a humidified atmosphere at 5% CO_2_ [37,38].

For the first and second experiments, cells were fixed with Transfix reagent (Cytomark, Buckingham, UK), following the company’s instructions [39] and were evaluated by serial analysis.

For the third experiment, PBMC sample was tested over time without adding fixative, but labeling with 7-AAD, a protein that identifies nonviable cells.

Samples were stained with CD30-PE (clone HRS4), CD45-FITC or CD45-KrO, CD3-FITC, and 7-AAD. FCM analysis was performed with a DxFlex flow cytometer and data were analyzed with Kaluza analysis software v. 2.1 (Beckman Coulter, Brea, CA, USA).

**Figure 10:**
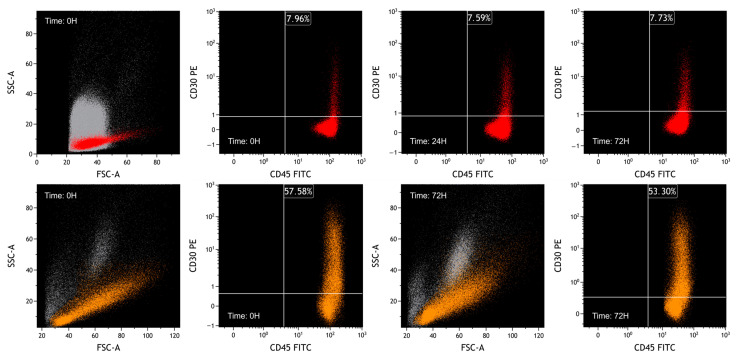
FCM plots of fixed-sample experiments. The grey population represents debris, mesothelial cells and leukocytes of ascitic fluid, the red population represents naturally activated lymphocytes, and the orange population represents PHA-activated PBMCs. Lymphocytes were selected based on FSC/SSC plot and CD45/SSC plot. For both situations, while characteristics of CD30 are similar over time, CD45 expression decreases, reflecting a reduction in MFI. In the lower plots, as previously observed, it can be seen that fixative can slightly alter the physical parameters.

In the plots of Figure 10, the results of fixed lymphocytes analyzed at time 0, after 24 and 72 h, are shown.

Nonviable cells were removed from the analysis by FSC/SSC plot.

We considered the median fluorescence intensity (MFI) over time, to observe the expression of CD30 in the studied cell populations. The autofluorescence was measured by acquiring an unmarked sample.

As we expected, the percentage of CD30-positive cells on naturally activated lymphocytes is lower than on PHA-activated PBMCs.

As illustrated in Table 2, substantial stability is documented in both the percentage of CD30-positive lymphocytes and in the CD30 MFI. In comparison, the variability of MFI values of the pan-leukocytic marker CD45 is much greater.

**Figure 11:**
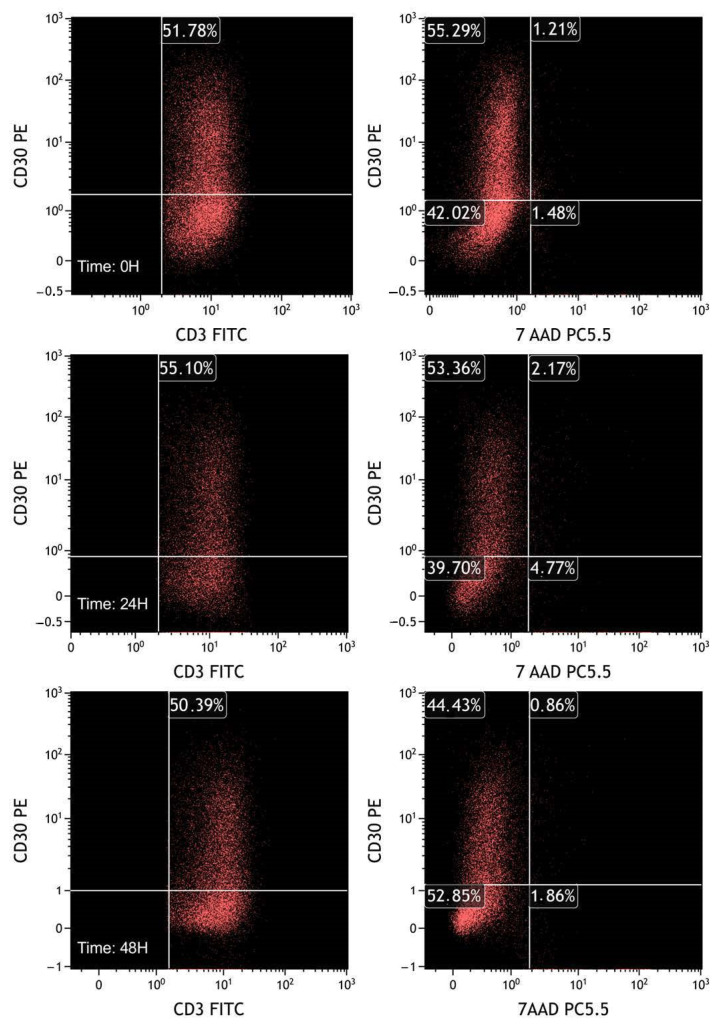
FCM plots of fresh sample experiments. T lymphocytes were selected based on CD3/CD45 plot gated on leukocytes, previously selected through the FSC/SSC plot. The plots on the left represent CD30 in T lymphocytes (CD3+). As in the previous experiments, the percentage of CD30 positive population is almost stable over time. On the right plots, CD30 expression is evaluated by dividing live (7-AAD negative) and dead (7-AAD positive) cells. It is notable that activated CD30 positive cells do not exhibit selective death compared with CD30 negative T lymphocytes.

In the third experiment, we incubated PBMCs with PHA (4 µg/mL) for 48 h and then labeled the cells with 7-AAD to evaluate the expression of CD30 in relation to cell viability (Figure 11 and Table 3).

Even within the limits of the time period (maximum 48 h from the first analysis), a selective death of CD30-positive cells could be excluded.

**Figure 12:**
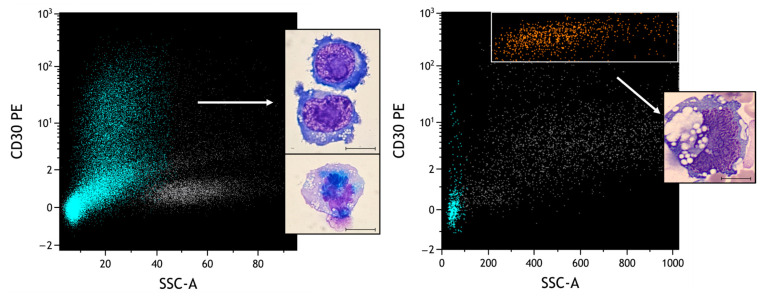
Comparison of different CD30-positive populations. Heavenly: T lymphocytes selected based on CD3/CD45 plot; orange: neoplastic cells; grey: remaining leukocyte quota. Under conditions of obvious atypical morphologies, the role of FCM is essential to distinguish reactive from neoplastic pictures. In particular, different SSCs and intensities of CD30 can be observed (MFI of activated lymphocytes: 2.39; MFI of neoplastic cells: 356). Scale bar: 10 µm.

Finally, we compared the morphology of PBMC lymphocytes stimulated with PHA (Figure 12, left plot) versus neoplastic lymphocytes from the second case of BIA-ALCL described (Figure 12, right plot).

It can be observed how, in the context of two frankly atypical morphologies, flow cytometric analysis can clearly distinguish the different intensities of expression of CD30. This information must be always integrated with the physical parameters and all the other surface markers typical of BIA-ALCL, and eventually other markers for the differential diagnosis.

## 5. Discussion

Cytological, IHC and flow cytometric analysis allow the diagnosis of BIA-ALCL.

Regarding morphology, in slides stained with Wright–Giemsa or May–Grünwald–Giemsa, one can see a varying number of lymphoid cells of different sizes, often large, atypical, or clearly anaplastic, with kidney-shaped or multinucleated nuclei resembling Reed–Sternberg cells, often with prominent nucleoli. The cytoplasm might be pale or variably basophilic, frequently containing many small vacuoles and sometimes showing irregular cell shapes, or cytoplasmic fragmentation. It is also common to observe apoptotic cells and atypical mitoses. The number of inflammatory cells in the background can vary, ranging from a few to a large number of small lymphocytes, neutrophils, histiocytes, or eosinophils. Necrotic tumor cells are frequently found [10,19].

When BIA-ALCL is suspected based on morphology [9,35], additional immunohistochemical markers are used, including T lineage markers (like CD2, CD7, CD5, CD3, CD4, and CD8) and cytotoxic markers (TIA-1, Granzyme B, and Perforin). CD30 is usually positive, while anaplastic lymphoma kinase (ALK-1) is always negative. However, this last characteristic alone doesn’t confirm a BIA-ALCL diagnosis since other systemic and cutaneous ALCL forms can also be ALK-negative [9]. Despite a normal morphology, the finding of rare CD30-positive lymphocytes does not require further investigation.

For differential diagnosis, it is crucial to rule out B-cell lymphomas, such as diffuse large B-cell lymphoma (for example, CD20, CD79a, PAX5, and EBER) [10,27].

A limitation of cytopathology and IHC analysis could be the difficulty in diagnosing BIA-ALCL in cases with minimal lymphomatous infiltration.

The NCCN recommends at least 50 mL of effusion (and as much as possible) for cytological and immunohistochemical examination [7,9]. Other studies indicate 20 mL volume as enough for the complete analyses [19]. A smaller amount of collected effusion, and repeated aspirations that dilute the number of cells, might compromise the ability to make a reliable diagnosis. In fact, especially when neoplastic cells are few, their distinction from non-cancerous cells can be a complex task. This limitation might be further enhanced by inter-observer variability, considering that IHC may be operator-dependent [40]. Therefore, the standardization of IHC can be challenging to avoid any possible misclassification [41].

Also for this reason, some studies view flow cytometric results as essential for diagnostic integration [33,34,35,36,42,43]. Cytometric analysis can differentiate the intensity of CD30 expression in various cell populations, allowing for simultaneous study with multiple labels.

In FCM, the neoplastic cells exhibit high light scatter similar to monocytes and/or granulocytes, likely due to the complexity of the nucleus and extensive cytoplasmic vacuolization. Typically, these cells fall outside the usual lymphocyte region and do not form distinct clusters on either the side light scatter versus forward light scatter plot or the side light scatter versus CD45 plot. This can make it difficult to identify the neoplasm when using gating strategies that primarily rely on physical parameters in conjunction with only CD45, especially in cases with relatively low percentages of cancerous cells.

The phenotype of this lymphoma is heterogeneous. The most frequent BIA-ALCL phenotype can be summarized as follows: CD2+ (sometimes weakly expressed), CD4+ (weak to moderate expression), CD30+ (bright expression), CD25+ (bright expression), HLA-DR+ (bright expression), CD45+ (weak expression or negative), surface CD3− (in most samples), CD7−, CD5−, and CD8− [35].

FCM is also useful to evaluate the intensity of CD30 and of the other markers, in order to identify different neoplastic subpopulations.

In our first case, we observed a bimodal expression of CD30 not associated with other phenotypic alterations. Other authors [43], however, have highlighted the potential presence of multiple clones, distinguishable phenotypically by the different expressions of various markers, including CD30.

Many BIA-ALCLs exhibit myeloid marker expression for CD15, CD13, and CD33. This may be related to the upregulation of genes involved in myeloid cell differentiation in BI-ALCL compared to ALK-negative systemic ALCL. Breast implant ALCL cases are negative for CD1a, TdT, and cyclin D1.

The proliferation index is typically very high (Ki-67 staining: 70–80% of the total cell count).

T-cell clonality, evaluated with FCM or PCR-based methods, confirms the T-cell origin of this neoplasm and appears of utility especially in the presence of uncertain morphological and phenotype features. However, molecular laboratories able to perform T-cell clonality are less available [10,14,25,44].

Infections, inflammation, or other lymphoproliferative disorders can simulate similar molecular findings, and this imposes a careful interpretation of clonality. The finding of clonality of the TCR in BIA-ALCL is variable in the various published studies [15,21,45].

With FCM, it is possible also to do a precise quantification of the types of leukocytes in the effusions. Generally, there is a lower percentage of neutrophils compared to lymphocytes and a predominance of CD3+ CD4+ T lymphocytes over CD3+ CD8+ T lymphocytes. B cells are usually scarce or entirely absent.

As described previously, CD30 plays a fundamental role in BIA-ALCL diagnosis.

CD30, belonging to the tumor necrosis factor receptor (TNF-R) superfamily, is a transmembrane protein consisting of 595 amino acids (105–120 kDa) [33,42].

It is present in various cell types, such as activated lymphocytes, monocytes, eosinophils, and mast cells.

CD30 expression is commonly seen in cells infected with viruses (HTLV-1, HIV-1, EBV) and it is overexpressed in lymphomas such as peripheral T-cell lymphoma, adult T-cell leukemia/lymphoma, NK lymphoma, and diffuse large B-cell lymphoma.

Actually, there is no specific threshold for the count of abnormal CD30+ cells in periprosthetic effusions needed to diagnose BI-ALCL. In most BIA-ALCL cases documented in scientific literature, CD30+ cancerous cells seem to constitute the majority of cells in the effusion. Non-cancerous effusions typically display a very small proportion of CD30+ cells, often less than 1% of the total cell count. Currently, there is an ambiguous zone where the percentage of CD30 cells is more than 1% but less than 10% [9,19].

An accurate evaluation of CD30 expression levels by FCM analysis might help in distinguishing different conditions. In fact, cytometric analysis can differentiate the intensity of CD30 expression in various cell populations, correlating it with the rest of the markers studied and the physical characteristics of the elements analyzed. It is essential to distinguish the expression between neoplastic cells (which have a greater intensity of CD30 expression) and reactive cells, to avoid false results [41,46,47].

However, FCM facilities are not available in all health centers, especially those that are small/medium-sized. It is not surprising, therefore, that in several country areas such as in Latin America, the diagnosis is usually based only on cytopathology and IHC [48]. To support the diagnostic work-up, specialized FCM laboratories might serve wide geographic areas by collecting from them samples after resolving the pre-analytical issue of sample stability.

In our experiments, albeit on a non-original matrix, we demonstrated that using a dedicated fixative avoids the death of CD30 positive cells.

Its use would allow any flow cytometric investigations to be carried out in the days following the collection of the material. The integration of flow cytometric with morphological results is also reiterated by the latest international NCCN guidelines of 2024. This data integration would allow for a better diagnosis, especially in more complex cases, and consequently a greater therapeutic chance.

We are aware that a limitation of our study is the use of no original matrix. However, by using PHA, we tried to simulate a state of strong cell activation and proliferation to obtain cells as similar as possible.

We observed these aspects in the morphological and cytofluorimetric characteristics of the experiment’s cells.

New examinations with BIA-ALCL cells will be necessary to confirm the data.

Pfreundschuh et al. [49] demonstrated generally higher serum CD30 levels in patients with CD30+ lymphoid neoplasms compared to patients with mononucleosis.

Kadin et al. [24] demonstrated that CD30+ neoplastic cells share some characteristics with Th1 and Th17 lymphocytes. BIA-ALCL cells produce IL-13 and IL-4 and the transcription factor GATA3, similar to Th2 lymphocytes. BIA-ALCL cell lines also secrete IL-13, IL-6, and IL-10 into tissue culture media.

The number of IL-13 transcripts seems to correlate with the number of anaplastic cells in BIA-ALCL. IL-13 also influences the tumor cell microenvironment and contributes to fibrosis, which is evident in thickened capsules affected by BIA-ALCL [50].

After binding to its ligand CD30L (CD153), CD30 triggers signals through tumor necrosis factor receptor-associated proteins (TRAFs), stimulating nuclear factor-kappa B (NFkB). Additionally, CD30 triggers signaling through *MAPK* pathways, including ERK1 and ERK2, which activate mitogen-activated protein kinase. Overall, CD30 plays a crucial role in cell survival, and for this reason it is also a potential target for therapy. In this regard, brentuximab vedotin is an anti-CD30 antibody conjugated to a cytotoxic agent that is already used in lymphomas’ therapy, while anti-CD30/CD16 antibody is another potential therapy. In preclinical studies on Hodgkin lymphoma, in fact, this bispecific antibody acts by creating an immunological synapse between CD30 positive cells and NK lymphocytes, with the consequent activation of NK cells and their release of substances such as granzyme B and Perforin on CD30+ cells resulting in their death [51].

## 6. Conclusions

Based on our experience, albeit in limited cases, we believe that flow cytometric study is essential for the diagnosis of BIA-ALCL together with cytology and IHC analyses. This concept becomes even more fundamental in cases where the neoplastic infiltrate is small.

Although on a non-original matrix, we demonstrated that a dedicated fixative does not alter the expression of CD30 on the cell surface and prevents selective death of these cells.

It remains essential that FCM correctly define the intensity of CD30 expression, considering it with a meticulous description of the physical parameters and all the other cellular markers studied with a detailed interpretative comment, to avoid false results between reactive and neoplastic cases.

Although further research is needed, cytofluorimetric description of the possible heterogeneity of CD30 expression on the lymphoma population could be useful to clinicians to explain possible resistance to target therapy.

## Figures and Tables

**Table 1:** Flow cytometric antigen expression and immunohistochemical stain of BIA-ALCL cases. mod: moderate; ND: not determined. EMA: epithelial membrane antigen; ALK-1: anaplastic lymphoma kinase-1; EBER: Epstein-Barr virus encoded small RNA, TIA-1: cytotoxic granule associated RNA binding protein.

FCM				IHC			
	Case 1	Case 2	Case 3		Case 1	Case 2	Case 3
Neoplastic cells	70%	50%	4%	Neoplastic cells	Numerous	Rare	ND
CD30	+ (mod to bright)	+ (bright)	+ (bright)	CD30	+	+	+
CD3	−	−	−	CD3	+ focal	−	−
CD4	+ (mod)	+	+	CD4	+ (focal/doubt)	+	+ (mod/doubt)
CD8	−	−	−	CD8	−	ND	−
CD2	+	−	−	CD2	+	ND	−
CD5	−	−	−	CD5	−	ND	ND
CD7	−	−	−	CD7	−	ND	+ (mod/doubt)
CD19	−	−	−	CD20	−	−	−
CD15	−	ND	ND	CD15	+ partial	+	+
CD56	−	−	−	AE1/AE3	−	ND	ND
CD16	−	−	−	EMA	+ partial	+	−
CD14	−	ND	ND	ALK-1	−	−	−
CD43	ND	+	ND	Perforin	+ (focal/doubt)	+	+ partial
CD45	−/+	+	+ (mod)	PAX5	−	−	−
HLA-DR	−	ND	ND	EBER	−	ND	ND
FSC	high	high	high	TIA-1	+ focal	ND	ND
SSC	high	high	high				

**Table 2:** Results of fixed-sample experiments. The percentage of CD30-positive cells on naturally activated lymphocytes is lower than on PHA-activated PBMCs. The percentage of CD30-positive cells is stable over time in fixed cells. In addition, we observed that the MFI of CD30 fluctuates visibly less than the MFI of CD45.

Experiments on Fixed Samples	Time of Acquisition (Hours)	Percentage of CD30+ Cells among Lymphocyte (%)	MFI of CD30	MFI of CD45
Lysed peripheral blood in ascitic fluid	0	7.96	2.39	136.23
	24	7.59	1.87	62.93
	72	7.73	2.51	47.40
PBMC stimulated with PHA in ascitic fluid	0	57.58	2.78	129.60
	72	53.30	1.38	39.74

**Table 3:** Results of fresh sample experiments. The percentage and MFI of CD30 positive cells are essentially stable after 24 and 48 h from collection of the sample, without adding fixative. On the basis of the positivity to 7-AAD, it is also observed that there is no selective death of CD30 positive cells.

Experiments on Fresh Samples	Time of Acquisition (Hours)	Percentage of CD30+ Cells (%)	MFI of CD30+ Cells	Percentage of CD30+/7AAD− Cells (%)	Percentage of CD30+/7AAD+ Cells (%)	Percentage of CD30−/7AAD+ Cells (%)
PBMC stimulated with PHA	0	51.78	7.79	55.29	1.21	1.48
	24	55.11	4.49	53.36	2.17	4.77
	48	50.39	4.06	44.43	0.86	1.86

## Data Availability

Data is contained within the article and Supplementary Materials.

## Supplementary Materials

The following supporting information can be downloaded at: https://www.mdpi.com/article/10.3390/ijms25063518/s1.
