# Supplementary material for: Flow Cytometry Analysis in Breast Implant-Associated Anaplastic Large Cell Lymphoma: Three Case Reports
Source: Int J Mol Sci. 2024 Mar 20;25(6):3518. doi: 10.3390/ijms25063518 (PMC10970634; doi:10.3390/ijms25063518)
Supplement: Supplementary file 1 [file ijms-25-03518-s001.zip › ijms-2901915-supplementary.pdf]

Table S1. Antibodies used in routine FCM analysis of periprosthetic fluid samples with suspected BIA-ALCL. The actual tube combination used for each sample was evaluated on a case-by-case basis. All antibodies are from Beckman Coulter Company (Brea, CA, USA).

Tube	FITC	PE	ECD	PE-Cy5	PE-Cy7	KrO
	Antibody (clone)	Antibody (clone)	Antibody (clone)	Antibody (clone)	Antibody (clone)	Antibody (clone)
1	CD4 (13B8.2)	CD30 (HRS4)	CD3 (UCHT1)	CD45 (J33)	-	
2	CD15 (80H5)	CD30 (HRS4)	CD3 (UCHT1)	CD45 (J33)	CD14 (RMO52)	
3	CD4 (13B8.2)	CD30 (HRS4)	CD3 (UCHT1)	CD45 (J33)	-	
4	CD2 (39C1.5)	CD4 (13B8.2)	CD3 (UCHT1)	CD7 (8H8.1)	CD5 (BL1a)	
5	CD45 (J33)	CD16/56 (3G8/N901)	CD19 (J3-119)	CD3 (UCHT1)	-	
6	CD45 (J33)	CD4 (13B8.2)	CD8 (SFCI21Thy2D3)	CD3 (UCHT1)	-	
7	CD2 (39C1.5)	CD30 (HRS4)	CD3 (UCHT1)	CD7 (8H8.1)	CD5 (BL1a)	
8	CD43 (DFT1)	CD30 (HRS4)	CD3 (UCHT1)	CD45 (J33)	CD4 (13B8.2)	
9	CD2 (39C1.5)	CD4 (13B8.2)	CD45 (J33)	HLA-DR (Immu-357)	-	
10	CD45 (J33)	CD30 (HRS4)	-	-	-	
11	CD3 (UCHT1)	CD30 (HRS4)		7-AAD		CD45 (J33)

Table S2. Antibodies used in routine IHC analysis of periprosthetic fluid samples with suspected BIA-ALCL. The actual stain combination used for each sample was evaluated on a case-by-case basis. Antibodies are from Leica (Milan, Italy), Dako (Milan, Italy), Biocare (Milan, Italy), Diagnostic Biosystems (Pleasanton, CA, USA).

IHC stain	Clone	Company	Antibody diluition
CD30	JCM182	Leica	pre-diluted
CD3	LN10	Leica	1:100
CD4	4B12	Dako	1:40
CD8	C8/144B	Dako	1:100
CD2	AB75	Dako	pre-diluted
CD5	4C7	Leica	1:200
CD7	LP15	Biocare	1:50
CD20	L26	Dako	1:200
CD15	MMA	Leica	pre-diluted
AE1/AE3	AE1/AE3	Leica	1:250
EMA	E29	Biocare	1:100
ALK-1	ALK-1	Dako	1:25
Perforin	5B10	Diagnostic Biosystems	1:10
PAX5	DAK-PAX5	Dako	1:100
TIA-1	TIA-1	Biocare	1:100
EBER	In situ hybridisation probe (Leica)		
